# Control of HIV-1 Replication by CD8^+^ T Cells Specific for Two Novel Pol Protective Epitopes in HIV-1 Subtype A/E Infection

**DOI:** 10.1128/jvi.00811-22

**Published:** 2022-09-26

**Authors:** Hung The Nguyen, Nozomi Kuse, Yu Zhang, Hayato Murakoshi, Yosuke Maeda, Yoshiko Tamura, Rie Maruyama, Giang Van Tran, Trung Vu Nguyen, Kinh Van Nguyen, Shinichi Oka, Takayuki Chikata, Masafumi Takiguchi

**Affiliations:** a Division of International Collaboration Research and Tokyo Joint Laboratory, Joint Research Center for Human Retrovirus Infection, Kumamoto University, Kumamoto, Japan; b Department of Microbiology, Faculty of Life Sciences, Kumamoto University, Kumamoto, Japan; c National Hospital of Tropical Diseases, Hanoi, Vietnam; d Hanoi Medical University, Hanoi, Vietnam; e AIDS Clinical Center, National Center for Global Health and Medicine, Tokyo, Japan; St. Jude Children’s Research Hospital

**Keywords:** protective epitopes, T cells, HIV-1 subtype A/E, escape mutation, HLA-B*15:02, CTL

## Abstract

Although many HIV-1-specific CD8^+^ T cell epitopes have been identified and used in various HIV-1 studies, most of these epitopes were derived from HIV-1 subtypes B and C. Only 17 well-defined epitopes, none of which were protective, have been identified for subtype A/E infection. The roles of HIV-1-specific T cells have been rarely analyzed for subtype A/E infection. In this study, we identified six novel HLA-B*15:02-restricted optimal HIV-1 subtype A/E epitopes and then analyzed the presentation of these epitopes by HIV-1 subtype A/E virus-infected cells and the T cell responses to these epitopes in treatment-naive HIV-1 subtype A/E-infected HLA-B*15:02^+^ Vietnamese individuals. Responders to the PolTY9 or PolLF10 epitope had a significantly lower plasma viral load (pVL) than nonresponders among HLA-B*15:02^+^ individuals, whereas no significant difference in pVL was found between responders to four other epitopes and nonresponders. The breadth of T cell responses to these two Pol epitopes correlated inversely with pVL. These findings suggest that HLA-B*15:02-restricted T cells specific for PolTY9 and PolLF10 contribute to the suppression of HIV-1 replication in HLA-B*15:02^+^ individuals. The HLA-B*15:02-associated mutation Pol266I reduced the recognition of PolTY9-specific T cells *in vitro* but did not affect HIV-1 replication by PolTY9-specific T cells in Pol266I mutant virus-infected individuals. These findings indicate that PolTY9-specific T cells suppress replication of the Pol266I mutant virus even though the T cells selected this mutant. This study demonstrates the effective role of T cells specific for these Pol epitopes to control circulating viruses in HIV-1 subtype A/E infection.

**IMPORTANCE** It is expected that HIV-1-specific CD8^+^ T cells that effectively suppress HIV-1 replication will contribute to HIV-1 vaccine development and therapy to achieve an HIV cure. T cells specific for protective epitopes were identified in HIV-1 subtype B and C infections but not in subtype A/E infection, which is epidemic in Southeast Asia. In the present study, we identified six T cell epitopes derived from the subtype A/E virus and demonstrated that T cells specific for two Pol epitopes effectively suppressed HIV-1 replication in treatment-naive Vietnamese individuals infected with HIV-1 subtype A/E. One of these Pol protective epitopes was conserved among circulating viruses, and one escape mutation was accumulated in the other epitope. This mutation did not critically affect HIV-1 control by specific T cells in HIV-1 subtype A/E-infected individuals. This study identified two protective Pol epitopes and characterized them in cases of HIV-1 subtype A/E infection.

## INTRODUCTION

HIV-specific cytotoxic T lymphocytes (CTLs) are critical components of the adaptive immune system that controls HIV-1 infection (1–7). Because these T cells recognize HIV-1-derived peptides presented by human leukocyte antigen (HLA) class I molecules on the surface of HIV-1-infected cells, the induction of HIV-1-specific CTLs and the role of these T cells in the control of HIV-1 replication are heterogeneous among populations with different HLA class I distributions and infected with different HIV-1 subtypes (8–12). HLA-B*57 and HLA-B*27 are protective alleles in Caucasian and African populations (12–16), whereas the HLA-B*52:01-C*12:02 haplotype and HLA-B*67:01 allele have a protective effect on clinical outcomes in Japan, where HLA-B*57 and HLA-B*27 are very rare (17). HIV-1-specific T cells restricted by these protective alleles contribute to the control of HIV-1 infection (1, 13, 18–22); therefore, the effect of protective HLA alleles in HIV-1 infection is predominantly related to T cells specific for these protective epitopes. Most of these protective HLA alleles and T cell epitopes were reported for subtype B and C infections (13, 14, 16, 17, 23–28), whereas limited numbers of protective HLA alleles and epitopes have been reported for other HIV-1 subtype infections.

HIV-1 subtype A/E (CRF01-AE) is predominantly found in Southeast Asia, including Thailand and Vietnam (29, 30). Progression to AIDS is more rapid after HIV-1 subtype A/E infection than after infection with other HIV-1 subtypes (31–33); however, its mechanism remains unknown. Few studies have investigated an association between HIV-1 disease progression and HLA alleles in HIV-1 subtype A/E infection. In previous analyses of small cohorts in Thailand, HLA-B*35:05 and HLA-B*51 were identified as protective alleles in terms of plasma viral load (pVL) and mortality, respectively (34, 35). A previous study of 536 treatment-naive Vietnamese individuals chronically infected with HIV-1 subtype A/E showed that HLA-C*12:02 was a protective allele that had a modest association with a good clinical outcome (low pVL and high CD4 T cell count), whereas HLA-A*29:01-B*07:05-C*15:05 was a detrimental haplotype (36). A recent study revealed that HLA-C*15:05 was associated with a detrimental effect in HIV-1 subtype A/E-infected Vietnamese individuals and that the accumulation of Pol S653A/T/L escape mutants impaired the recognition of HLA-C*15:05-restricted SL9-specific CTLs and led to a poor clinical outcome in HIV-1-infected Vietnamese individuals carrying the HLA-C*15:05 allele (37).

The number of reported HIV-1-specific CTL epitopes has increased to more than 2,000, including 280 well-defined epitopes (best-defined CTL epitopes) in the Los Alamos HIV-1 database (https://www.hiv.lanl.gov). Among these epitopes or epitope candidates, approximately 1,200 and 550 were reported for subtype B and C infections, respectively, whereas approximately 140 were reported for subtype A/E infection. Similarly, most of the best-defined CTL epitopes were reported for subtype B and C infections, and only 17 best-defined epitopes have been identified in subtype A/E infection. Furthermore, protective epitopes have not been reported for HIV-1 subtype A/E infection. This small number of reported well-defined subtype A/E epitopes has restricted the studies of T cell immunology, immunopathogenesis, and vaccine development related to subtype A/E infection. Therefore, it is critical to identify these epitopes.

In the present study, we sought to identify optimal CD8^+^ T cell epitopes and protective ones in subtype A/E infection. We first identified responders to each peptide cocktail showing lower pVL or higher CD4 than nonresponders. These T cells were most frequently found in HLA-B*15:02^+^ individuals among those harboring each HLA-B allele. We therefore sought to identify HIV-1-specific HLA-B*15:02-restricted epitopes and then investigated whether T cells specific for these epitopes suppressed HIV-1 replication in subtype A/E infection.

## RESULTS

### T cell responses to 17-mer overlapping peptides covering HIV-1 subtype A/E Nef, Gag, and Pol.

**FIG 1:**
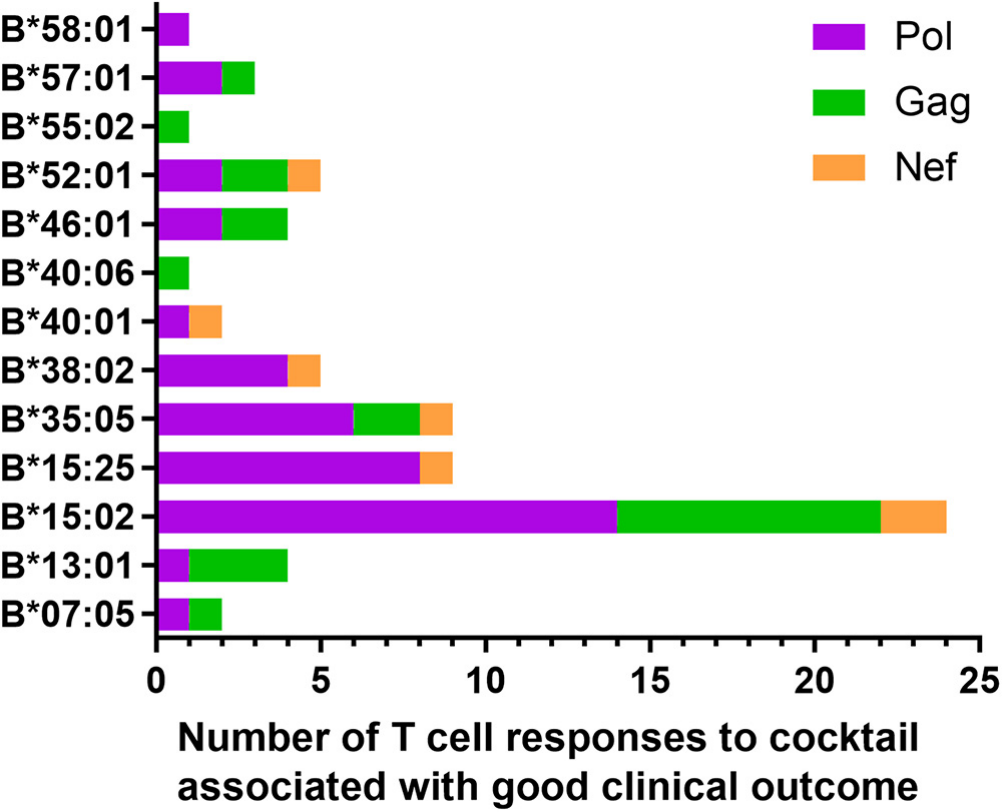
Number of T cell responses to HIV-1 peptide cocktails associated with a good clinical outcome in patients harboring each HLA-B allele. The number of T cell responses to HIV-1 peptide cocktails associated with a good clinical outcome (pVL or CD4 T cell count) is presented for each HLA-B allele. When responders to the HIV-1 peptide cocktail had a significantly lower pVL or higher CD4 count than nonresponders among HIV-1 subtype A/E-infected individuals harboring each HLA-B allele, the T cell responses were evaluated for their association with a good clinical outcome.

We investigated T cell responses to 17-mer overlapping peptides covering the consensus sequences of HIV-1 subtype A/E Nef, Gag, and Pol proteins among 395 treatment-naive people living with HIV-1 (PLWH) in Vietnam. We generated 281 17-mer peptides and divided them into 35 peptide cocktail groups (8 or 9 17-mer peptides in each peptide cocktail group). We investigated T cell responses to these peptide cocktails (4 Nef, 10 Gag, and 21 Pol peptide cocktails) by performing *ex vivo* enzyme-linked immunosorbent spot (ELISpot) assays. Because previous studies showed that protective HIV-1 epitopes were predominantly found in HLA-B alleles (15, 24–28), we focused on HLA-B allele-restricted T cell responses in the current study. We analyzed the differences in clinical outcomes (pVL and CD4 T cell count) between responders and nonresponders to each peptide cocktail among individuals harboring each HLA-B allele and then identified T cell responses in responders with a significantly lower pVL or higher CD4 count than that of nonresponders. Among individuals harboring each HLA-B allele, we found 70 T cell responses matched these criteria (Fig. 1). Among individuals harboring each HLA allele, HLA-B*15:02^+^ individuals had the highest number of these T cell responses (Fig. 1). These findings imply that protective HIV-1-specific T cells are more frequent in HLA-B*15:02^+^ individuals than in individuals harboring other HLA-B alleles in HIV-1 subtype A/E-infected Vietnamese. There are no reports of HLA-B*15:02-restricted epitopes in HIV-1 subtype A/E infection. Therefore, we focused on HLA-B*15:02^+^ individuals when identifying protective epitope-specific T cells.

### HLA-B*15:02-restricted CD8^+^ T cell responses to 17-mer peptides.

**FIG 2:**
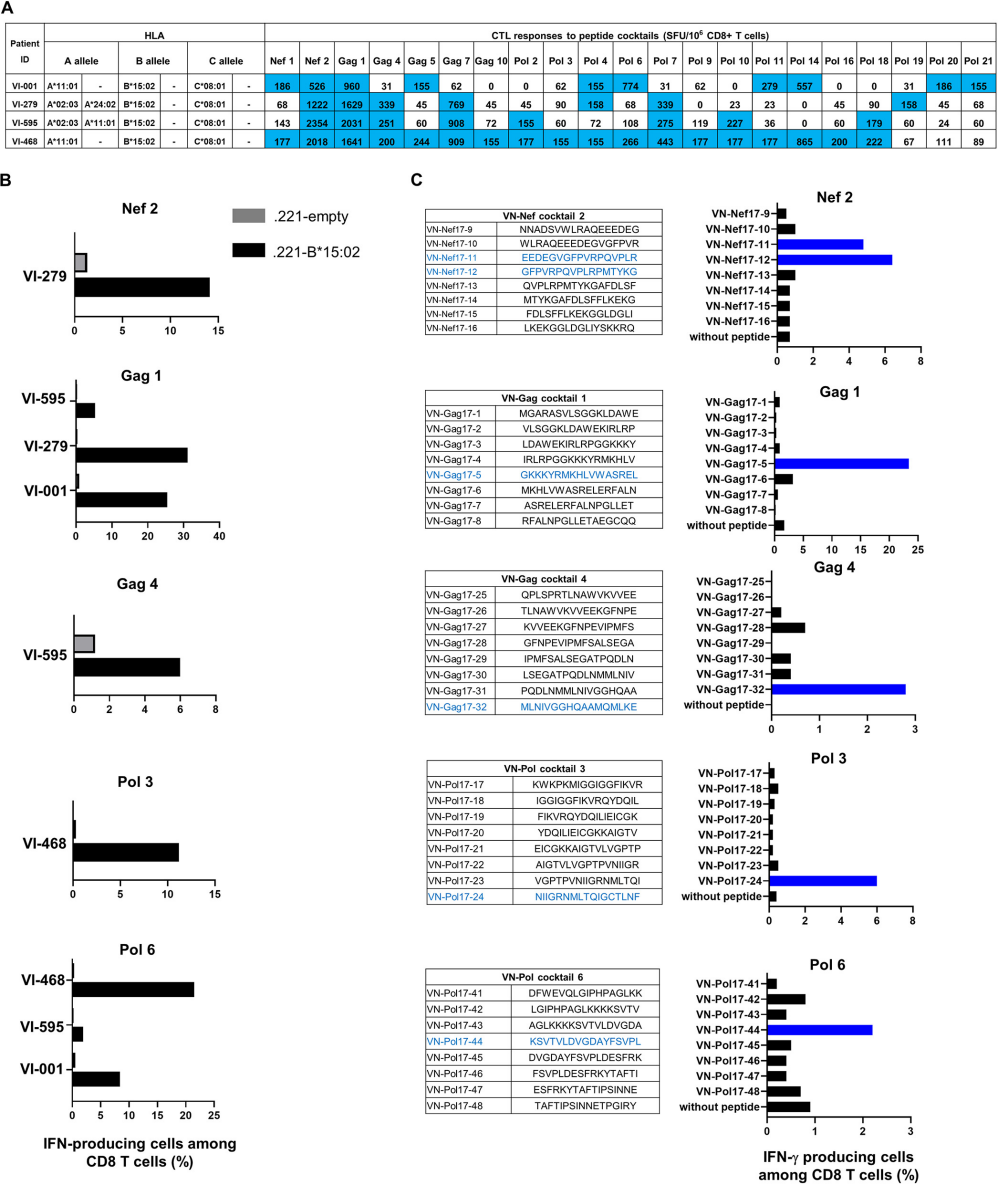
HLA-B*15:02-restricted CD8^+^ T cell responses to 17-mer cocktails and single peptides. (A) T cell responses to 17-mer overlapping cocktails. T-cell responses in PBMCs derived from 4 HIV-1 subtype A/E-infected Vietnamese individuals were analyzed by IFN-γ ELISpot assays. A positive response (≥150 SFU/10^6^ CD8^+^ T cells) is indicated in blue. (B) HLA-B*15:02-restricted CD8^+^ T cell responses to five 17-mer overlapping peptide cocktails. Responses of bulk T cells derived from 4 individuals stimulated with peptide cocktails (Nef2, Gag1, Gag4, Pol3, and Pol6) to .221-B*15:02 and .221 cells prepulsed with the peptide cocktail at a 1 μM peptide concentration were analyzed by ICS assay. (C) HLA-B*15:02-restricted CD8^+^ T cell responses to 17-mer overlapping single peptides. Responses of bulk T cells to .221-B*15:02 cells prepulsed with 17-mer overlapping single peptides in each cocktail at a concentration of 1 μM were analyzed by ICS assay. Bulk T cells specific for Nef2, Gag4, and Pol3 were derived from VI-279, VI-595, and VI-468, respectively, and bulk T cells established from VI-001 were used for the analysis of Gag1 and Pol6.

To identify HIV-1-specific HLA-B*15:02-restricted T cell responses, we selected four HLA-B*15:02 homozygote individuals who showed positive T cell responses to 7 to 18 HIV-1 peptide cocktails (>150 spots) in an *ex vivo* ELISpot assay (Fig. 2A). We stimulated the peripheral blood mononuclear cells (PBMCs) of these individuals with the peptide cocktails and cultured them for 12 to 14 days. Responses of these bulk T cells to the peptide cocktails were tested by performing an intracellular cytokine staining (ICS) assay using 721.221 cells expressing only HLA-B*15:02 (.221-B*15:02) prepulsed with the peptide cocktails. We found HLA-B*15:02-restricted T cell responses to five cocktails (Nef2, Gag1, Gag4, Pol3, and Pol6) in these individuals (Fig. 2B).

Next, we determined the HLA-B*15:02-restricted T cell recognition of single peptides included in each peptide cocktail. Nef2-specific T cells responded to 2 single peptides, VN-Nef17-11 and VN-Nef17-12, whereas Gag1-specific, Gag4-specific, Pol3-specific, and Pol6-specific T cells recognized VN-Gag17-5, VN-Gag17-32, VN-Pol17-24, and VN-Pol17-44, respectively (Fig. 2C). These results indicate that these 17-mer peptides include HLA-B*15:02-restricted T cell epitopes.

### Identification of optimal HLA-B*15:02-restricted HIV-1 subtype A/E epitopes.

**FIG 3:**
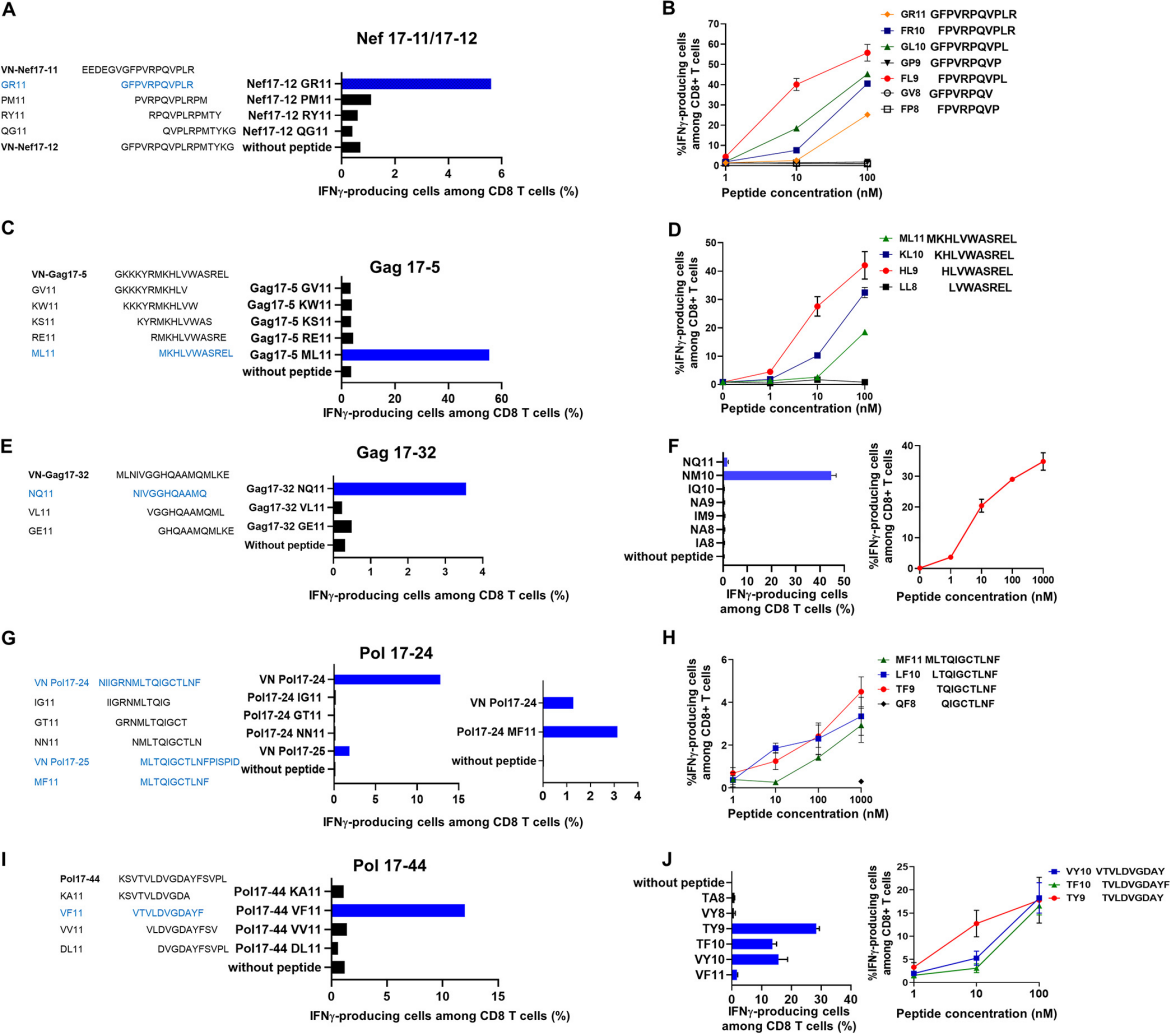
Identification of optimal HLA-B*15:02-restricted T cell epitopes. T cell responses to .221-B*15:02 cells prepulsed with 11-mer peptides covering the 17-mer peptides (A, C, E, G, and I) and those with truncated peptides (B, D, F, H, and J) were analyzed by ICS assay. (A) Responses of Nef17-11/17-12-specific T cells (VI-279) to four 11-mer peptides spanning VN-Nef17-12 at a concentration of 1 μM. (B) Responses of NefGR11-specific T cells (VI-279) to 6 truncated peptides from 1 to 100 nM. (C) Responses of Gag17-5-specific T cells (VI-001) to five 11-mer peptides spanning VN-Gag17-5 at a concentration of 1 μM. (D) Responses of GagML11-specific T cells (VI-001) to 4 truncated peptides from 1 to 100 nM. (E) Responses of Gag17-32-specific T cells (VI-114) to three 11-mer peptides spanning VN-Gag17-32 at a concentration of 1 μM. (F) (Left) Responses of GagNQ11-specific T cells (VI-114) to 7 truncated peptides at a concentration of 1 μM. (Right) Responses of GagNM10-specific T cells (VI-114) to NM10 peptide from 1 to 1,000 nM. (G). (Left) Responses of Pol17-24-specific T cells (VI-114) to three 11-mer peptides spanning VN-Pol17-24 and VN-Pol17-25 at a concentration of 1 μM. (Right) Responses of Pol17-24-specific T cells (VI-114) to MF11 peptide at a concentration of 1 μM. (H) Responses of PolMF11-specific T cells (VI-114) to 4 truncated peptides from 1 to 1,000 nM. (I) Responses of Pol17-44-specific T cells (VI-001) to four 11-mer peptides spanning VN-Pol17-44 at a concentration of 1 μM. (J) (Left) Responses of Pol17-44-specific T cells (VI-001) to 6 truncated peptides at a concentration of 1 μM. (Right) Responses of Pol17-44-specific T cells (VI-001) to 3 truncated peptides from 1 to 100 nM. Data shown in panels B, D, F, H, and J were from assays performed in triplicate.

Next, we identified HLA-B*15:02-restricted optimal epitope peptides in these single 17-mer peptides. We generated a panel of 11-mer peptides spanning the sequence of these 17-mer peptides and investigated whether they were recognized by the bulk T cells. Nef2-specific T cells recognized VN-Nef17-11 and especially VN-Nef17-12 peptides (Fig. 2C). Therefore, we synthesized four 11-mer peptides covering VN-Nef17-12 and tested their recognition by Nef2-specific T cells. Nef2-specific T cells responded only to VN-Nef17-12 GR11 (Fig. 3A). Then, we synthesized 8-mer to 10-mer peptides covering VN-Nef17-12 GR11 and tested their recognition by Nef2-specific T cells. The T cells recognized NefFL9 (FPVRPQVPL) to a higher degree than they recognized the other truncated peptides (Fig. 3B), indicating that NefFL9 is an HLA-B*15:02-restricted T cell epitope.

To identify Gag epitopes recognized by Gag17-5-specific and Gag17-32-specific T cells, we synthesized 11-mer peptides covering VN-Gag17-5 and VN-Gag17-32 and analyzed their recognition by these T cells. Gag17-5-specific T cells recognized VN-GagML11 and Gag17-32-specific T cells recognized VN-GagNQ11 (Fig. 3C and E). Because Gag17-5-specific T cells failed to recognize VN-GagRE11, it is likely that Leu at the C terminus of the peptide is necessary for the T cell recognition. Then, we synthesized truncated 8-mer to 10-mer peptides with Leu at the C terminus and tested their recognition by Gag17-5-specific T cells. We found that Gag17-5-specific T cells recognized GagHL9 peptide to a greater degree than they recognized other peptides (Fig. 3D), indicating that GagHL9 is an optimal T cell epitope. We also synthesized six truncated 8-mer to 10-mer peptides within the VN-GagNQ11 peptide. Gag17-32-specific T cells recognized only the GagNM10 peptide (Fig. 3F). Thus, GagHL9 and GagNM10 are HLA-B*15:02-restricted T cell epitopes.

To identify Pol epitopes recognized by Pol17-24-specific and Pol17-44-specific T cells, we generated three 11-mer peptides covering Pol17-24 and four 11-mer peptides covering VN-Pol17-44 and tested their recognition by Pol17-24-specific and Pol17-44-specific T cells. Pol17-24-specific T cells failed to recognize all three 11-mer peptides but did weakly recognize VN-Pol17-25 (Fig. 3G, left). This suggested that the T cells recognized peptides with Phe at the C terminus. Therefore, we synthesized the MF11 peptide and tested its recognition by Pol17-24-specific T cells. Indeed, Pol17-24-specific T cells recognized MF11 to a greater degree than they recognized the VN-Pol17-24 peptide (Fig. 3G, right). Because Phe at the C terminus is necessary for the recognition of this epitope, we synthesized 8-mer to 10-mer peptides with Phe at the C terminus and investigated their recognition by Pol17-24-specific T cells. The T cells recognized TF9 and LF10 at similar levels and to greater degrees than they recognized other truncated peptides (Fig. 3H). These results suggest that PolTF9 and PolLF10 are recognized as superimposed peptides. Pol17-44-specific T cells recognized only the VF11 peptide (Fig. 3I). Therefore, we synthesized 8-mer to 10-mer truncated peptides within the VF11 peptide and investigated their recognition by Pol17-44-specific T cells. The T cells recognized the TY9, TF10, and VY10 peptides to a greater degree than they recognized VF11 (Fig. 3J, left). Finally, we analyzed the T cell recognition of titrated peptides and demonstrated that the T cells recognized TY9 to a greater degree than they recognized two 10-mer peptides (Fig. 3J, right), indicating that PolTY9 was the optimal epitope.

### Recognition of HIV-1-infected cells by HLA-B*15:02-restricted CD8^+^ T cells specific for 6 epitopes.

**FIG 4:**
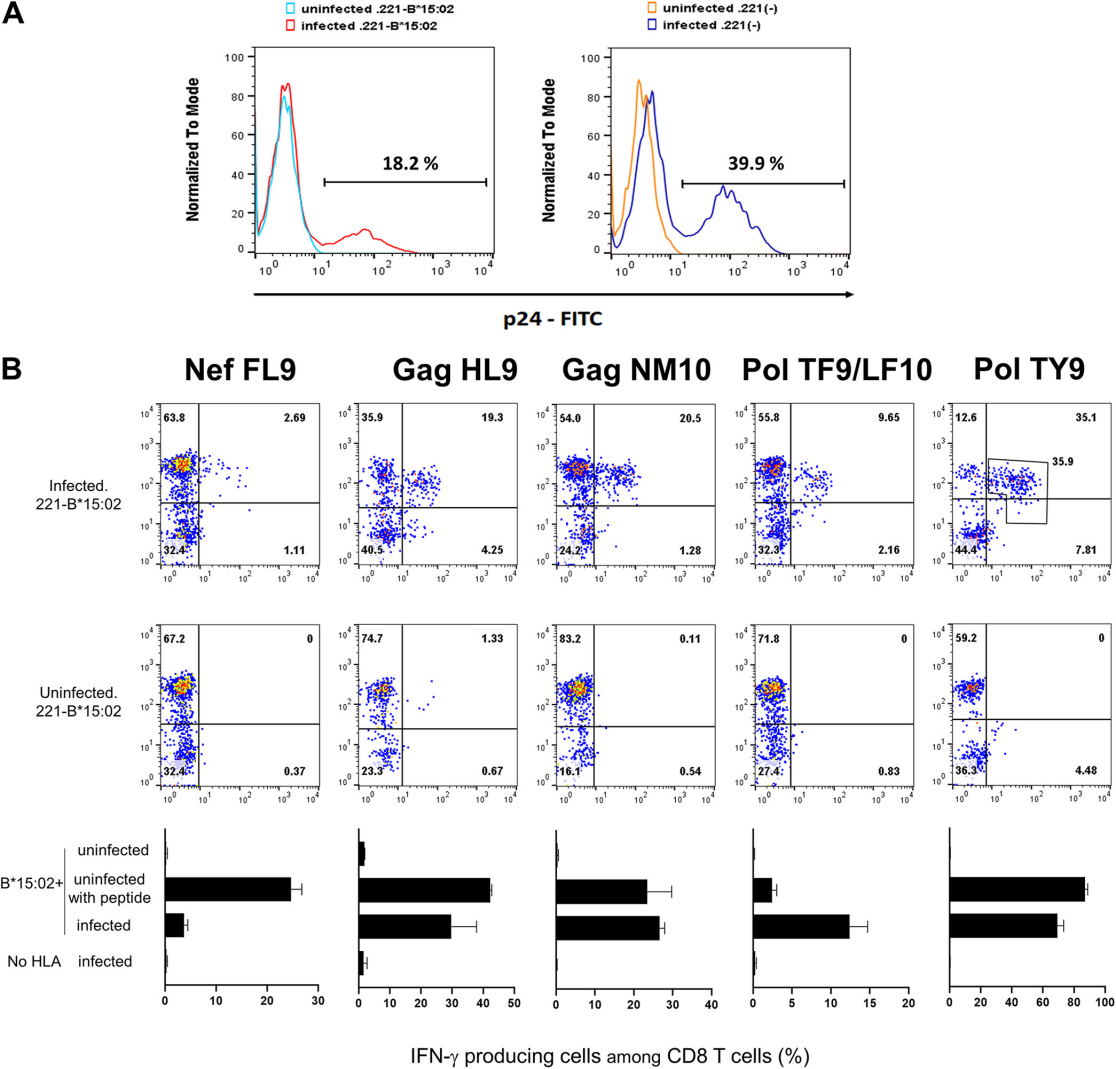
Recognition of HIV-1 subtype A/E-infected cells by CD8^+^ T cells specific for 5 HLA-B*15:02-restricted epitopes. (A) Histogram plots showing the frequency of p24 antigen-positive cells among VI-157X4 virus-infected .221-B*15:02 cells or VI-157X4 virus-infected .221 cells. FITC, fluorescein isothiocyanate. (B) The responses of bulk T cells specific for NefFL9, GagHL9, GagNM10, PolTF9/LF10, or PolTY9 to VI-157X4 virus-infected .221-B*15:02 cells were analyzed by ICS assay. The bulk T cells shown in Fig. 3 were used for this analysis. The frequencies of p24 antigen-positive cells among VI-157X4-infected .221-B1502 cells and VI-157X4-infected .221 cells in each analysis of epitope-specific CD8^+^ T cells were 18.2% and 39.9% for PolTY9-specific T cells, 19.8% and 37.6% for GagHL9- and GagNM10-specific T cells, and 19.4% and 37.0% for NefFL9- and PolTF9/LF10-specific T cells, respectively. The top two rows display representative data for the percentage of IFN-γ production from epitope-specific CD8^+^ bulk T cells with HIV-1-infected and uninfected .221-B*15:02 cells. The results in the bottom row are the means and SD of assays performed in triplicate.

To investigate the antigen presentation ability of 6 epitopes in HIV-1 subtype A/E virus-infected cells, we isolated HIV-1 subtype A/E viruses from our Vietnam cohort (38) and then generated a molecular clone derived from the X4 viruses (VI-157X4). .221-B*15:02 and .221 cells were infected with VI-157X4 (Fig. 4A). Then, we investigated the ability of the HLA-B*15:02-restricted T cells to recognize HLA-B*15:02^+^ cells infected with HIV-1 subtype A/E by performing an ICS assay using VI-157X4-infected .221-B*15:02 cells and VI-157X4-infected .221 cells. T cells specific for NefFL9, GagHL9, GagNM10, PolTF9/LF10, or PolTY9 recognized VI-157X4-infected .221-B*15:02 cells but not VI-157X4-infected .221 cells (Fig. 4B). These results indicate that these HIV-1 epitopes were presented by HLA-B*15:02 on the HIV-1-infected cells.

### HLA-B*15:02-restricted immunodominant epitopes.

**FIG 5:**
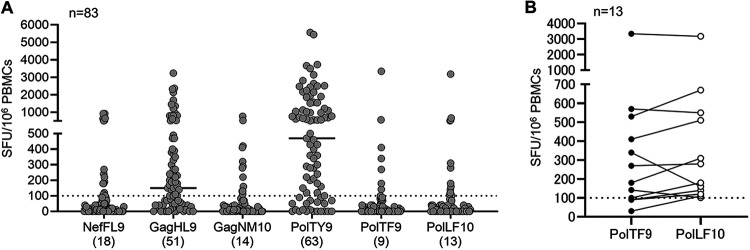
Immunogenicity of HLA-B*15:02-restricted epitopes in HIV-1 subtype A/E infection. (A) T cell responses to 6 HLA-B*15:02-restricted epitopes in 83 HLA-B*15:02^+^ Vietnamese individuals infected with HIV-1 subtype A/E. Responses to peptides at a concentration of 1 μM were analyzed by *ex vivo* IFN-γ ELISpot assay. Horizontal bars in graphs represent the median number of spots. The number of responders is shown below each epitope label. (B) T cell responses to superimposed epitopes of PolTF9 and PolTF10 in the same HLA-B*15:02^+^ individuals. T cell responses to PolTF9 and PolLF10 were compared between 13 responders to PolTF9 or PolLF10. The dotted line at 100 SFU/10^6^ PBMCs indicates the threshold for a positive response.

Next, we investigated the frequency of responders to these 5 epitopes in 83 treatment-naive HLA-B*15:02^+^ individuals infected with HIV-1 subtype A/E. Because PolTF9 and PolLF10 were evaluated as superimposed peptides (Fig. 3H), we included LF10 peptide in the analysis. T cell responses to 6 epitope peptides were tested using PBMCs isolated from 83 individuals by *ex vivo* ELISpot assay. Responses to each peptide were detected in >10% of individuals tested (Fig. 5A). Responses to PolTY9 and GagHL9 were found in 75.9% and 61.4% of the individuals tested, respectively. These results indicate that PolTY9 and GagHL9 are strong immunodominant epitopes. Responses to TF9 and LF10 were detected in 9 and 13 individuals, respectively. The magnitudes of these responses were similar in these individuals, although different patterns of T cell recognition for these 2 peptides were found (Fig. 5B). These findings suggest that they are presented as superimposed epitopes at similar levels in these individuals.

### Variation of 5 HLA-B*15:02-restricted epitopes among circulating subtype A/E viruses in Vietnamese individuals.

We analyzed the HIV-1 sequences of 6 HLA-B*15:02-restricted epitopes among 380 treatment-naive PLWH in Vietnam. The top three most frequent sequences corresponding to these epitopes are shown in Table 1. More than 75% of the individuals had wild-type (WT) sequences for NefFL9, GagHL9, GagNM10, and PolTY9. The WT sequence of GagNM10 was found in 95% of individuals, while the WT sequences of NefFL9, PolTY9, and GagHL9 were found in 80.0%, 87.6%, and 77.1% of individuals, respectively. In contrast, the WT sequence of PolTF9/LF10 was found in only 62.4% of individuals. The variant sequence of PolTF9-3L was found in 31% of individuals. Three variant sequences, NefFL9-4K, GagHL9-2I, and PolTY9-5I, were detected in 11.8%, 10.3%, and 6.18% of individuals, respectively. Thus, 4 HLA-B*15:02-restricted epitopes were relatively conserved among the circulating viruses.

**TABLE 1:** HIV-1 sequences corresponding to 6 epitopes among Vietnamese individuals chronically infected with HIV-1 subtype A/E

Epitope and variant	HXB2 position	Sequence^*a*^	No. positive/total no. (frequency [%])
Nef FL9	Nef68-76	FPVRPQVPL	304/380 (80.0)
Nef FL9-4K		FPV**K**PQVPL	45/380 (11.84)
Nef FL9-9V		FPVRPQVP**V**	4/380 (1.05)
Gag HL9	Gag33-41	HLVWASREL	277/359 (77.15)
Gag HL9-2I		H**I**VWASREL	37/359 (10.30)
Gag HL9-3I		HL**I**WASREL	13/359 (3.62)
Gag NM10	Gag189-198	NIVGGHQAAM	341/359 (94.98)
Gag NM10-8G		NIVGGHQ**G**AM	6/359 (1.67)
Gag NM10-3I		NI**I**GGHQAAM	4/359 (1.11)
Pol TF9	Pol147-155	TQIGCTLNF	231/370 (62.43)
Pol TF9-3L		TQ**L**GCTLNF	116/370 (31.35)
Pol TF9-3V		TQ**V**GCTLNF	5/370 (1.35)
Pol LF10	Pol146-155	LTQIGCTLNF	231/370 (62.43)
Pol LF10-4L		LTQ**L**GCTLNF	116/370 (31.35)
Pol LF10-4V		LTQ**V**GCTLNF	5/370 (1.35)
Pol TY9	Pol262-270	TVLDVGDAY	326/372 (87.63)
Pol TY9-5I		TVLD**I**GDAY	23/372 (6.18)
Pol TY9-1S		**S**VLDVGDAY	15/372 (4.03)

a The mutated amino acid is in bold.

### Recognition of variant epitopes by HLA-B*15:02-restricted HIV-1-specific CD8^+^ T cells.

**FIG 6:**
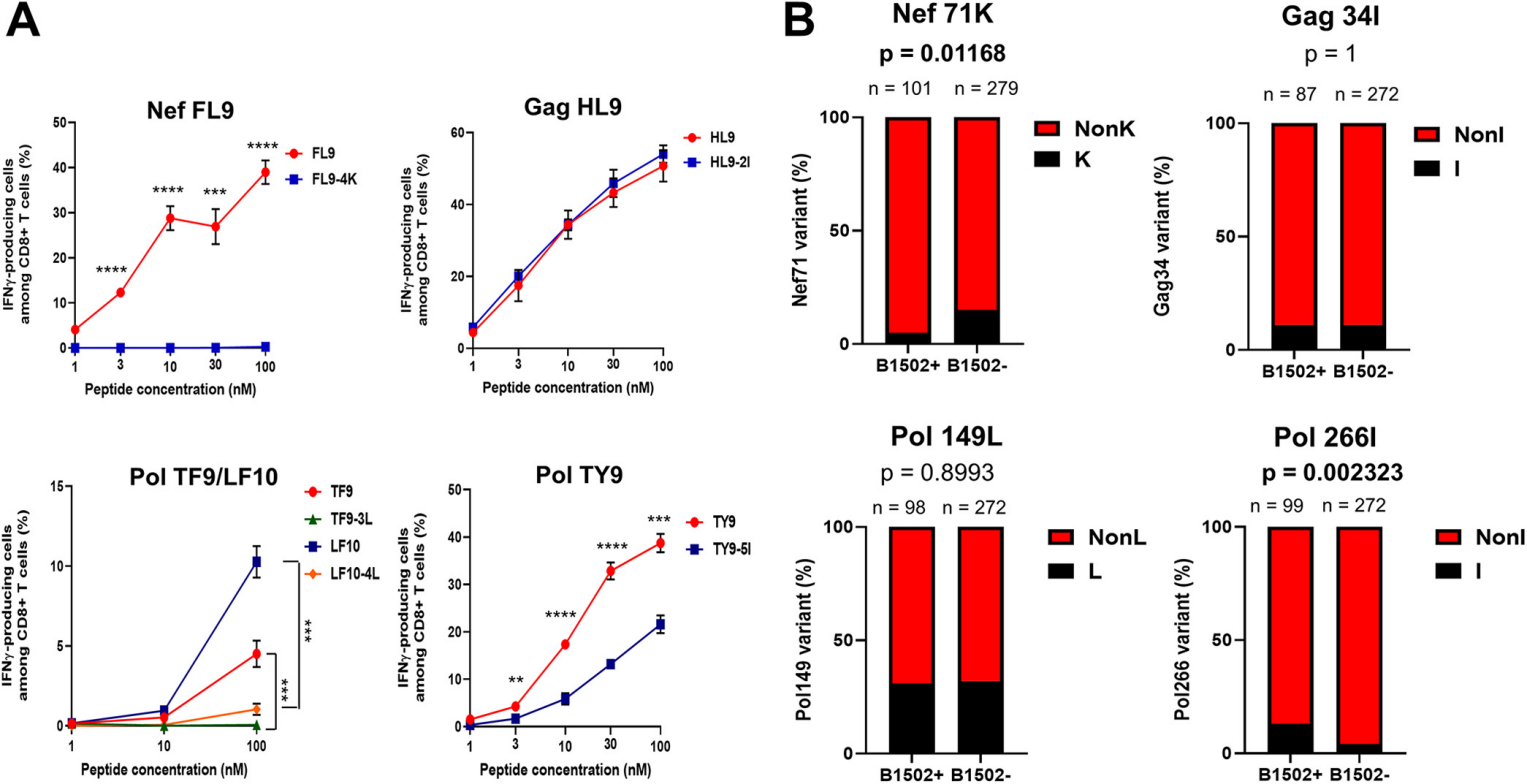
Recognition of mutant epitope peptides by CD8^+^ T cells specific for 4 epitopes. (A) The responses of bulk T cells specific for 4 epitopes (NefFL9, GagHL9, PolTF9/LF10, and PolTY9) to .221-B*15:02 cells prepulsed with wild-type or mutant peptides at different concentrations were analyzed by ICS assay. The results are shown as the means and SD from assays performed in triplicate. Statistical analysis was performed by unpaired *t* test. **, *P* < 0.01; ***, *P* < 0.001; ****, *P* < 0.0001. (B) Association of 4 mutations (Nef71K, Gag34I, Pol149L, and Pol266I) with HLA-B*15:02. The frequency of mutations between HLA-B*15:02^+^ and HLA-B*15:02^−^ individuals was analyzed statistically by Fisher’s exact test.

To investigate the recognition of variant peptides in circulating HIV-1 by wild-type-specific T cells, we selected and generated variant peptides that were detected in >5% of the individuals tested. We analyzed 5 mutant peptides (NefFL9-4K, GagHL9-2I, PolTF9-3L/LF10-4L, and PolTY9-5I) corresponding to 5 HLA-B*15:02-restricted epitopes (Fig. 6A). The NefFL9-specific T cells failed to recognize the NefFL9-4K mutant peptide, whereas GagHL9-specific T cells cross-recognized wild-type and GagHL9-2I mutant peptides at similar levels. PolTF9/LF10-specific T cells failed to recognize PolTF9-3L/LF10-4L peptides, whereas the recognition of PolTY9-5I peptide by PolTY9-specific T cells was significantly lower than that for the WT peptide. These findings indicate that these 3 mutations in Nef and Pol may affect the recognition of NefFL9-specific, PolTF9/LF10-specific, and PolTY9-specific T cells.

Next, we analyzed the sequences of these epitopes in HLA-B*15:02^+^ individuals (Table 2). We compared these epitope sequences between total and HLA-B*15:02^+^ individuals and found similar patterns of wild-type and mutant epitope sequences. The frequencies of WT sequences were similar for GagNM10, GagHL9, and PolTF9/LF10 between total and HLA-B*15:02^+^ individuals, whereas the frequencies of WT sequences in NefFL9 and PolTY9 were higher and lower in HLA-B*15:02^+^ individuals, respectively, than those in total individuals. These findings imply that these mutations include HLA-B*15:02-associated mutations. Therefore, we analyzed the association of four mutations (Nef71K, Gag34I, Pol149L, and Pol266I) with HLA-B*15:02. The frequency of Pol266I was significantly higher in HLA-B*15:02^+^ individuals than in HLA-B*15:02^–^ individuals (Fig. 6B), indicating that the Pol266I mutation had accumulated in HLA-B*15:02^+^ individuals. This is consistent with a previous study reporting HLA-associated polymorphisms in HIV-1 subtype A/E-infected Vietnamese individuals (39). The frequency of the Nef71K mutation was significantly lower in HLA-B*15:02^+^ individuals than in HLA-B*15:02^–^ individuals (Fig. 6B), suggesting that this mutation was not selected by HIV-1-specific T cells restricted by HLA-B*15:02.

**TABLE 2:** HIV-1 sequences corresponding to 6 epitopes among HLA-B*15:02^+^ Vietnamese individuals chronically infected with HIV-1 subtype A/E

Epitope and variant	HXB2 position	Sequence^*a*^	No. positive/total no. (frequency [%])
Nef FL9	Nef68-76	FPVRPQVPL	87/101 (86.14)
Nef FL9-4K		FPV**K**PQVPL	5/101 (4.95)
Nef FL9-9V		FPVRPQVP**V**	2/101 (1.98)
Gag HL9	Gag33-41	HLVWASREL	65/87 (74.71)
Gag HL9-2I		H**I**VWASREL	8/87 (9.20)
Gag HL9-3I		HL**I**WASREL	3/87 (3.45)
Gag NM10	Gag189-198	NIVGGHQAAM	83/87 (95.4)
Gag NM10-8G		NIVGGHQ**G**AM	1/87 (1.15)
Gag NM10-3I		NI**I**GGHQAAM	1/87 (1.15)
Pol TF9	Pol147-155	TQIGCTLNF	59/98 (60.20)
Pol TF9-3L		TQ**L**GCTLNF	30/98 (30.61)
Pol TF9-3V		TQ**V**GCTLNF	2/98 (2.04)
Pol LF10	Pol146-155	LTQIGCTLNF	59/98 (60.20)
Pol LF10-4L		LTQ**L**GCTLNF	30/98 (30.61)
Pol LF10-4V		LTQ**V**GCTLNF	2/98 (2.04)
Pol TY9	Pol262-270	TVLDVGDAY	75/100 (75.00)
Pol TY9-5I		TVLD**I**GDAY	13/100 (13.00)
Pol TY9-1S		**S**VLDVGDAY	7/100 (7.00)

a The mutated amino acid is in bold.

**FIG 7:**
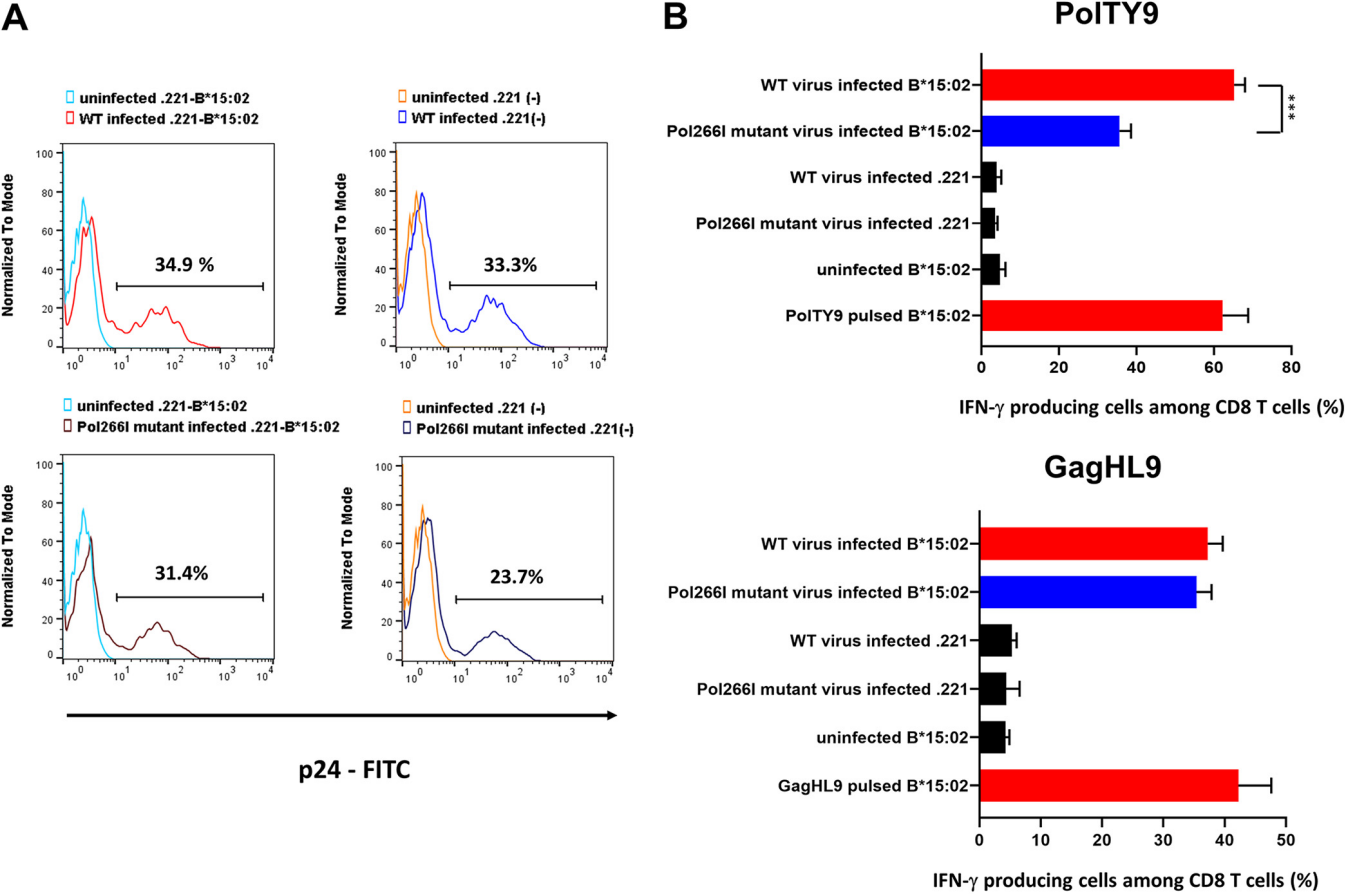
Recognition of Pol266I mutant virus-infected cells by PolTY9-specific T cells. (A) Histogram plots showing the frequency of p24 antigen-positive cells among VI-157X4 virus (WT)-infected or Pol266I mutant virus-infected .221-B*15:02 cells or .221 cells. (B) Responses of PolTY9-specific T cells or GagHL9-specific T cells to .221-B*15:02 cells or .221 cells infected with VI-157X4 virus or Pol266I mutant virus .221-B*15:02 cells pulsed with PolTY9 peptide or GagHL9 peptide (1,000 nM) were used as a positive control for the target cells. Statistical analysis of T cell responses to VI-157X4 virus-infected and Pol266I mutant virus-infected. 221-B*15:02 cells was performed using an unpaired *t* test. ***, *P* < 0.001.

The recognition of PolTY9-5I peptide by HLA-B*15:02-restricted PolTY9-specific T cells was significantly lower than that of the WT peptide (Fig. 6A), implying that Pol266I mutation affects the recognition of Pol226I mutant virus-infected cells by PolTY9-specific T cells in individuals infected with this mutant virus. To analyze the recognition of Pol266I mutant virus-infected cells by PolTY9-specific T cells, we generated Pol226I mutant virus from VI-157X4 subtype A/E virus and then infected 221-B*15:02 and .221 cells with VI-157X4 (WT virus) or Pol266I mutant virus (Fig. 7A). Then, we examined the ability of the PolTY9-specific T cells to recognize HLA-B*15:02^+^ cells infected with Pol266I mutant virus or WT virus. The PolTY9-specific T cells effectively recognized the WT virus-infected cells and, to a significantly lower extent, the Pol266I mutant virus-infected cells (Fig. 7B, top). HLA-B*15:02-restricted GagHL9-specific T cells recognized these target cells at similar levels (Fig. 7B, bottom). These results indicate that the Pol266I mutation reduced the recognition of Pol226I mutant virus-infected cells by PolTY9-specific T cells. However, this reduction in recognition may not be critical for the suppression of this mutant virus because the PolTY9-specific T cells can still recognize Pol266I mutant virus-infected cells.

### Association of T cell responses to HLA-B*15:02 epitope peptides with clinical outcomes.

**FIG 8:**
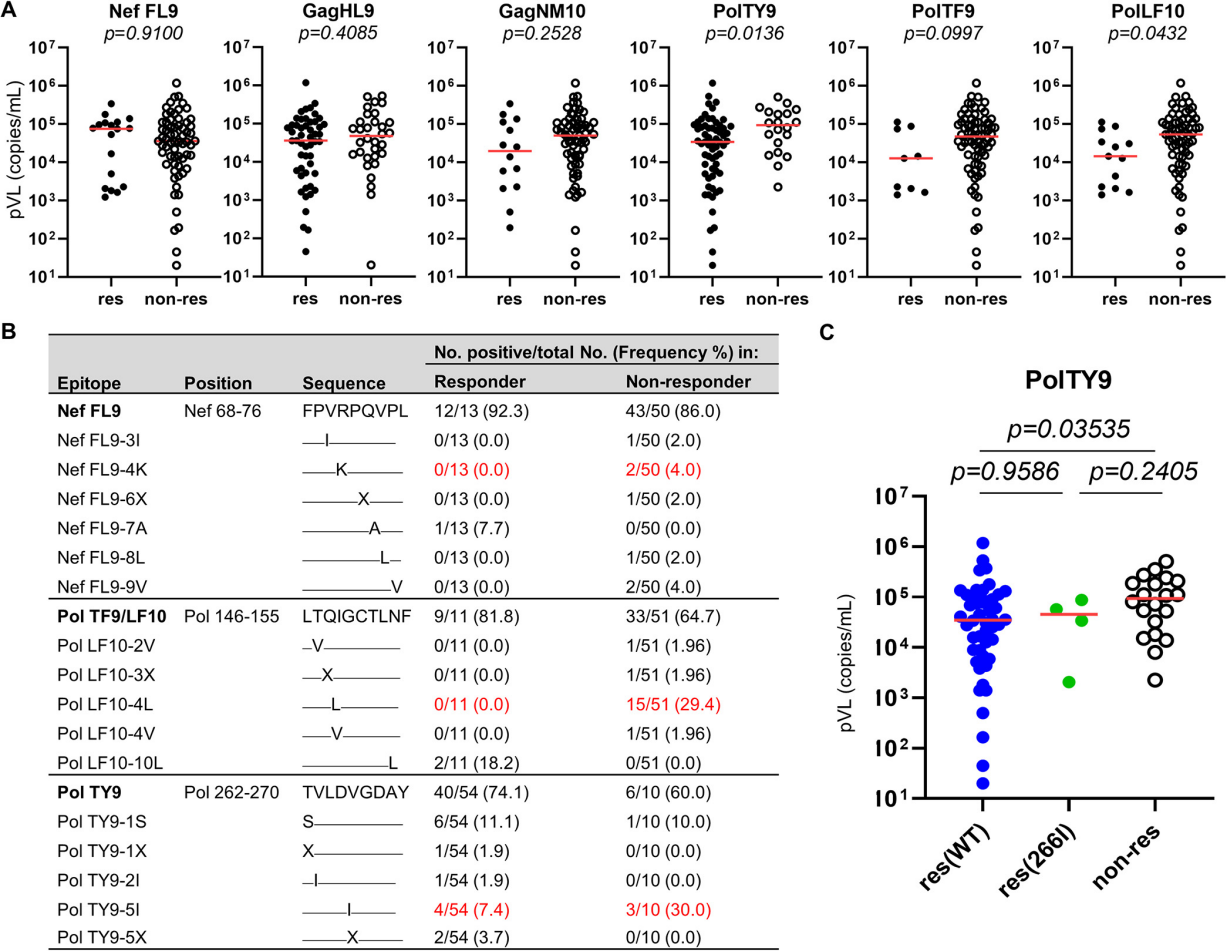
Effect of HLA-B*15:02-restricted CD8^+^ T cells on the clinical outcome of HIV-1-infected HLA-B*15:02^+^ individuals. (A) Association of T cell responses to HLA-B*15:02-restricted epitopes with pVLs. T cell responses to peptides at a concentration of 1 μM in 83 HLA-B*15:02^+^ Vietnamese individuals infected with HIV-1 subtype A/E were analyzed by *ex vivo* IFN-γ ELISpot assay. (B) Frequencies of HIV-1 sequences corresponding to 4 epitopes (NefFL9, PolTF9/LF10, and PolTY9) among responders and nonresponders. Boldface indicates a conserved epitope. (C) Comparison of pVL between responders to PolTY9 in wild-type virus-infected individuals (*n* = 48), those in Pol266I mutant virus-infected individuals (*n* = 4), and nonresponders (*n* = 20). The red lines in each figure represent the median pVL. Statistical analysis was performed using the Mann-Whitney U test (A and C).

We analyzed the association of T cell responses to HLA-B*15:02-restricted epitope peptides with clinical outcomes in 83 treatment-naive HLA-B*15:02^+^ individuals infected with the subtype A/E virus. Responders to PolTY9 or PolLF10 had a significantly lower pVL than nonresponders (Fig. 8A), suggesting that PolTY9 and PolLF10 are protective epitopes. Because the 3 mutations (Nef71K, Pol149L, and Pol266I) in Nef and Pol epitopes affected T cell recognition *in vitro* (Fig. 6A), we searched for accumulation of these mutations in the responders and nonresponders. Nef71K and Pol149L were detected only in the nonresponders, whereas Pol266I was found in the responders and nonresponders (Fig. 8B). The responders to PolTY9 were divided into 2 groups, responders with the WT sequence and those with Pol266I. Then, pVLs were compared between the responder and nonresponder groups. Responders with the Pol266I mutation showed a trend toward a lower pVL than that of nonresponders, whereas no significant difference in pVL was found between responders harboring Pol266I and those with the WT sequence (Fig. 8C), implying that PolTY9-specific T cells might suppress the replication of Pol266I mutant virus in HLA-B*15:02^+^ individuals infected with the Pol266I mutant virus.

**FIG 9:**
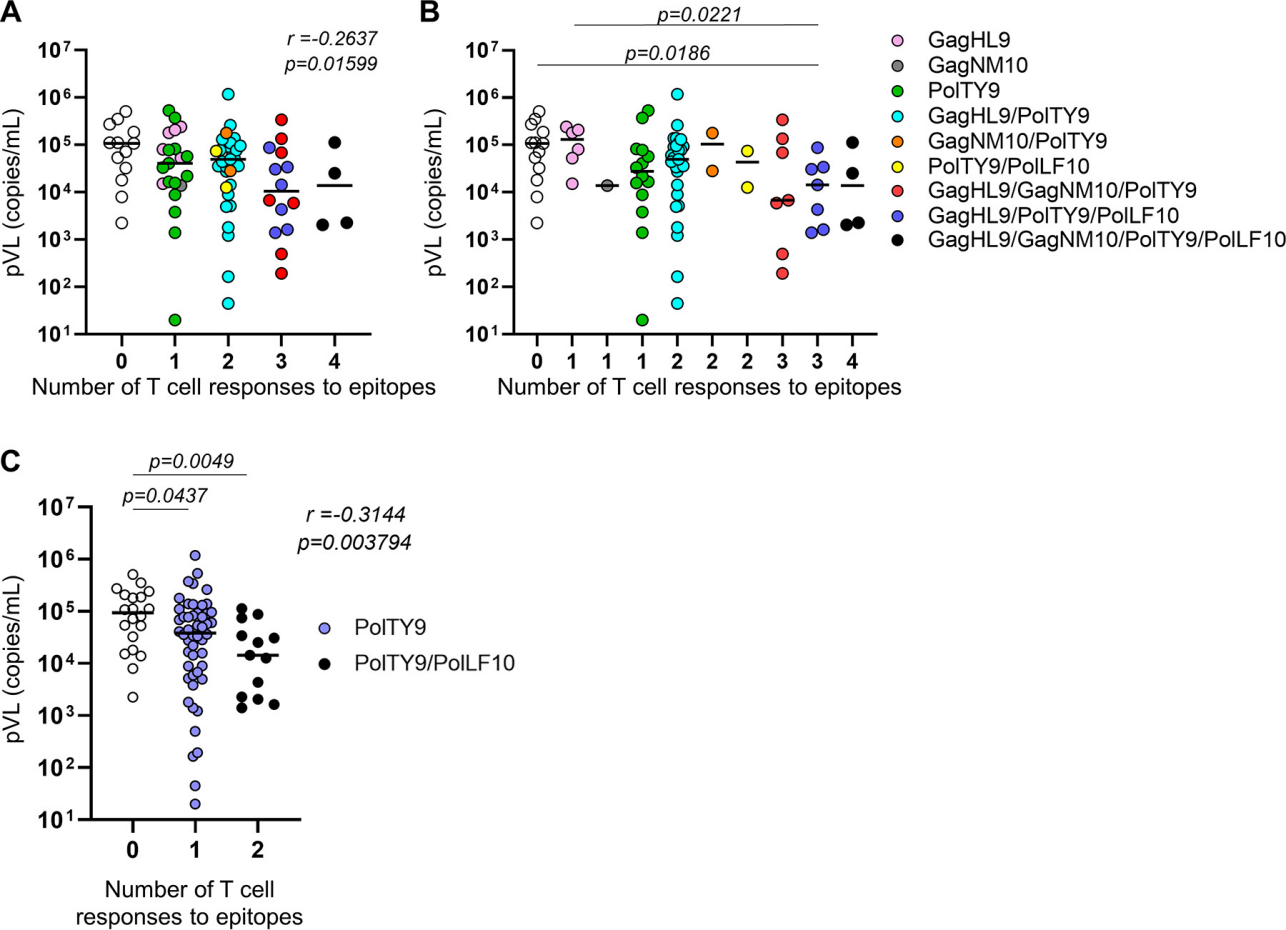
Collaboration of T cells specific for 4 HLA-B*15:02-restricted epitopes or 2 HLA-B*15:02-restricted Pol epitopes in the suppression of HIV-1 replication. (A) Correlation between the breadth of T cell responses to four HLA-B*15:02-restricted epitopes (GagHL9, GagNM10, PolTY9, and PolLF10) and pVL. (B) Correlation between T cell responses to a combination of HLA-B*15:02-restricted epitopes and pVL. (C) Correlation between the breadth of T cell responses to 2 HLA-B*15:02-restricted Pol epitopes (PolTY9 and PolLF10) and pVL. The lines in each panel represent the median pVL. Statistical analysis was performed using Spearman’s rank correlation test (A and C) and the Mann-Whitney U test (B and C).

Finally, we analyzed the correlation between the pVL and the breadth of T cell responses to the 4 HLA-B*15:02-restricted epitopes (GagHL9, GagNM10, PolTY9, and PolLF10) or to two Pol epitopes. The breadth of T cell responses to the 4 epitopes was inversely and significantly correlated with the pVL (Fig. 9A). We also analyzed the correlation between T cell responses to a combination of HLA-B*15:02-restricted epitopes and pVL. A significant correlation was found between the nonresponders and responders to GagHL9/PolTY9/PolLF10 and between responders to GagHL9 and responders to GagHL9/PolTY9/PolLF10 (Fig. 9B), suggesting that T cells specific for two Pol epitopes may suppress HIV-1 replication *in vivo*. Indeed, a correlation was found between the breadth of responses to two Pol epitopes and pVL (*r* = −0.3144; *P* = 0.003794) and between responders to two Pol epitopes and nonresponders (Fig. 9C). These results suggest that T cells specific for the two Pol epitopes have a predominant role in the suppression of HIV-1 replication in Vietnamese individuals infected with the subtype A/E virus.

## DISCUSSION

Because CD8^+^ T cells specific for protective HIV-1 epitopes can effectively suppress HIV-1 replication *in vivo*, it is expected that the identification of protective T cell epitopes will contribute to the development of AIDS vaccines and HIV-1 cures. Most protective T cell epitopes have been identified in HIV-1 subtype B and C infections, whereas the identification of protective epitopes in subtype A/E infections has not been reported. A previous study of Vietnamese individuals infected with HIV-1 subtype A/E showed that only HLA-C*12:02 was associated with a low pVL and high CD4 count (36). This implies that most protective epitopes are also presented by HLA class I alleles other than HLA-C*12:02 in HIV-1 subtype A/E-infected Vietnamese individuals. Furthermore, protective HIV-1-specific T cell responses were predominantly restricted by the HLA-B allele in HIV-1 subtype B and C infections (15, 24–28). Therefore, the current study attempted to identify protective epitopes presented by HLA-B in a Vietnamese cohort infected with subtype A/E. We identified T cell responses associated with good clinical outcomes in individuals carrying each HLA-B allele and found that HLA-B*15:02^+^ individuals had the highest number of T cell responses to peptide cocktails associated with a good clinical outcome. Then, we focused on the identification of HLA-B*15:02-restricted protective T cell epitopes. Responses to the peptide cocktails in HLA-B*15:02^+^ individuals detected by *ex vivo* ELISpot assay included HIV-1-specific HLA-B*15:02-restricted T cells as well as HIV-1-specific T cells restricted by other many HLA class I alleles. Therefore, we analyzed HLA-B*15:02 homozygote individuals to identify HIV-1-specific HLA-B*15:02-restricted T cells. Although we found that T cell responses to 24 peptide cocktails were associated with a good clinical outcome, we identified only 6 HLA-B*15:02-restricted epitopes in 5 peptide cocktails from 4 HIV-1 subtype A/E-infected individuals homozygous for HLA-B*15:02. This discrepancy might be explained as follows: T cell responses to some peptide cocktails might be restricted by HLA-A*11:01 or HLA-C*08:01 because the HLA-A*11:01-B*15:02-C*08:01 haplotype has a strong linkage disequilibrium in HIV-1-infected Vietnamese individuals (40).

In the current study, we identified 6 novel HLA-B*15:02-restricted epitopes (NefFL9, GagHL9, GagNM10, PolTF9/LF10, and PolTY9) in HIV-1 subtype A/E infection. A previous study showed that T cell responses to PolTY9 were significantly associated with HLA-B*15:02 in a cohort of individuals infected with subtype B, implying that PolTY9 might be an HLA-B*15:02-restricted epitope in subtype B infection (41). The current study definitively demonstrated that PolTY9 is an HLA-B*15:02-restricted epitope in subtype A/E infection. Out of 17 reported best-defined epitopes derived from the subtype A/E virus (Los Alamos HIV-1 database [https://www.hiv.lanl.gov]), 5 epitopes only were analyzed to examine their presentation by cells infected with HIV-1 subtype A/E in our previous studies (42–44). In this study, we demonstrated that T cells specific for HLA-B*15:02-restricted epitopes recognized cells infected with an HIV-1 subtype A/E clone established from an individual in our Hanoi cohort. These findings confirmed that these 6 epitopes were presented in Vietnamese individuals infected with subtype A/E viruses.

The Pol266I mutation was positively associated with HLA-B*15:02, strongly suggesting that this mutation was selected by HLA-B*15:02-restricted PolTY9-specific T cells and accumulated in HLA-B*15:02^+^ individuals. The *in vitro* analysis of PolTY9-specific T cells demonstrated that their recognition of the PolTY9-5I peptide was approximately 5 times lower than that of the wild-type peptide, whereas there was no difference in the pVLs between responders to the PolTY9 peptide who were infected with the Pol266I mutant virus and those who were infected with the wild-type virus. These findings suggest a minimal effect of this escape mutation on the ability of PolTY9-specific T cells to suppress the replication of the Pol266I mutant virus in HLA-B*15:02^+^ individuals infected with this virus. Indeed, PolTY9-specific T cells effectively recognized the Pol266I mutant virus-infected cells, although their recognition of WT virus-infected cells was greater. In contrast, Nef71K was negatively associated with HLA-B*15:02. A previous study of HLA-associated polymorphisms in HIV-1 subtype A/E-infected Vietnamese individuals reported an association of Nef71K with HLA-C*07:02/B*07:05/B*15:12 (39), suggesting that this mutation was selected by HIV-1-specific T cells restricted by these HLA alleles. The Nef71K mutation critically affected the recognition of NefFL9-specific T cells *in vitro*, whereas T cells were not elicited in HLA-B*15:02^+^ individuals infected with this mutant virus. These findings suggest that the accumulation of this mutation critically affected the induction of NefFL9-specific T cells. Similarly, PolTF9/LF10-specific T cells failed to recognize PolTF9-3L (LF10-4L) mutant peptides and T cells were not elicited in HLA-B*15:02^+^ individuals infected with this mutant virus. These results suggest that the Pol149L mutation affects the induction of PolTF9/LF10-specific T cells. A previous study reported an association between Pol149L and HLA-B*15:12 (39), suggesting that this mutation was selected by HLA-B*15:12-restricted T cells. Nef71K and Pol149L might be transmitted into HLA-B*15:02^+^ individuals or selected by T cells restricted by HLA-C*07:02/B*07:05/B*15:12 in HLA-B*15:02^+^ individuals harboring these HLA alleles. These preadaptive mutations may affect the induction of NefFL9-specific and PolTF9/LF10-specific T cells in HIV-1-infected HLA-B*15:02^+^ individuals. Indeed, Pol149L and Nef71K mutations were found only in HLA-B*15:02^+^ nonresponders. Taken together, these findings suggest that the accumulation of Nef71K and Pol149L mutant viruses selected by T cells restricted by HLA-C*07:02/B*07:05/B*15:12 critically affected the induction of specific T cells in HLA-B*15:02^+^ individuals.

Although Gag-specific CD8^+^ T cells have a greater ability to suppress HIV-1 than do Pol-specific CD8^+^ T cells in HIV-1 subtype B and C infections (45–47), several protective Pol epitopes were identified in subtype B infections (7, 22, 28, 48). In the current study, we showed that Pol-specific CD8^+^ T cells suppressed HIV-1 replication compared with Gag-specific CD8^+^ T cells in HIV-1 subtype A/E-infected HLA-B*15:02^+^ individuals, although whether Pol-specific CD8^+^ T cells contribute to the control of HIV-1 infection more than Gag-specific CD8^+^ T cells in subtype A/E infections remains unclear. Because only two protective epitopes in subtype A/E infections have been identified, the identification of other protective CD8^+^ T cell epitopes is required to clarify the role of Pol-specific CD8^+^ T cells in HIV-1 subtype A/E infections.

Several studies reported that the progression to AIDS after HIV-1 subtype A/E infection was more rapid than that after infection by other HIV-1 subtypes (31–33). However, the mechanism(s) involved remains unknown. A previous study demonstrated that the transmission of viruses preadapted to HLA molecules expressed in a recipient was associated with impaired immunogenicity, elevated viral load, and accelerated CD4^+^ T cell decline (49). The accumulation of preadapted mutations in protective epitopes might explain the rapid progression of HIV-1 subtype A/E infection. The identification of other protective epitopes and analyses using these epitopes in HIV-1 subtype A/E infection are required to clarify the mechanism(s) involved in the rapid progression.

Here, we report two protective CD8^+^ T cell Pol epitopes in subtype A/E infections. However, it is still difficult to determine the effects of HIV-1-specific T cells on HIV-1 control in subtype A/E infection because limited numbers of protective T cell epitopes have been reported for subtype A/E infections. The identification of other HIV-1 subtype A/E protective epitopes will help determine the role of T cells specific for these protective epitopes in HIV-1 control in cases of subtype A/E infection. In addition, it is expected that these protective epitopes will contribute to the development of HIV-1 vaccines and HIV cures.

## MATERIALS AND METHODS

### Subjects and ethics statement.

Overall, 395 treatment-naive Vietnamese individuals chronically infected with subtype A/E were recruited in the National Hospital of Tropical Diseases (NHTD), Hanoi, Vietnam. The study protocol was approved by the Ethics Committee of the Vietnamese Ministry of Health (1666/QĐ-BYT) and by the Ethics Committees of Kumamoto University (RINRI-1340 and GENOME-342). Informed consent was obtained from all individuals according to the Declaration of Helsinki. pVLs were measured by Cobas TaqMan HIV-1 real-time PCR (Roche Diagnostics, NJ, USA).

### HLA genotyping.

HLA-A, -B, and -C genotypes were identified by the Luminex microbead method (Luminex 100 system; Luminex Corporate, Austin, TX, USA) at the NPO HLA Laboratory (Kyoto, Japan). They were reported according to the nomenclature of The HLA Dictionary (50).

### Peptides.

17-mer overlapping peptides spanning Nef, Gag, and Pol of HIV-1 subtype A/E consensus sequences and truncated peptides (8- to 11-mer) were synthesized using an automated multiple-peptide synthesizer and purified by high-performance liquid chromatography (HPLC). These 17-mer peptides overlapped by 11 amino acids. Thirty-five peptide cocktails were generated by mixing 8 or 9 peptides per cocktail (4 for Nef, 10 for Gag, and 21 for Pol) for *ex vivo* ELISpot assays. The purity (>90%) was examined using HPLC and mass spectrometry.

### Cell lines.

721.221 cells expressing CD4 molecules and HLA-B*15:02 (.221-B*15:02) were generated by transfecting the human CD4 gene and HLA-B*15:02 gene into 721.221 cells as previously described (51). These cells were maintained in RPMI 1640 medium containing 5% fetal bovine serum (FBS), 0.15 mg/mL hygromycin B (Calbiochem, Darmstadt, Germany), and 0.1 mg/mL kanamycin.

### Sequencing of plasma HIV-1 RNA.

HIV-1 RNA was extracted from plasma samples using a QIAmp UltraSens virus kit (Qiagen, Valencia, CA, USA) and then used for two rounds of PCR. Sequences were analyzed and aligned with the subtype A/E sequence as previously described (39). Sequences have been deposited in the DDBJ/EMBL/GenBank under accession numbers LC100161 to LC100529 (Gag), LC100902 to LC101260 and LC728341 to LC728351 (Pol), and LC100530 to LC100901 and LC728352 to LC728359 (Nef).

### Isolation of HIV-1 subtype A/E and construction of full-length infectious molecular clone VI-157X4.

HIV-1 subtype A/E was isolated from the plasma of a treatment-naive HIV-1^+^ individual collected at the NHTD as previously described (39). To construct a full-length infectious molecular HIV-1 clone from the isolated virus, a single HIV-1 clone was established using the SupT1 cell line by limiting dilution of the virus. DNA from the infected cells was then extracted and the entire genome was amplified in two fragments using Platinum SuperFi II DNA polymerase (Thermo Fisher Scientific, Vilnius, Lithuania). The 5′ fragment extended from the 5′ long terminal repeat (LTR) to the *vif* region, and the 3′ fragment extended from the *vif* region to the 3′ LTR. The 5′ fragment was amplified using the forward primer 5′-TGGATGGGCTAGTTTACTCCAAGAAAAGGAAAGAG-3′ and reverse primer 5′-GTCGGTGCTTCCGCTTCTTTCTGCCATAGG-3′, and the 3′ fragment was amplified using the forward primer 5′-CAGGGACAGCAGAGACCCAATTTGGAAAGG-3′ and reverse primer 5′-TGCTAGAGATTTTTACTCAGTCTAGAGTGGTCTGAGGG-3′. The amplified products were then cloned into a pCR-TOPO vector (Thermo Fisher Scientific) and sequenced using an ABI Prism 3130 automated sequencer (Thermo Fisher Scientific). The 5′ HIV-1 fragment was excised by restriction enzyme NotI and shared PflMI sites in the *vif* region and inserted into the 3′ HIV-1 fragment, thus generating the full-length infectious molecular clone VI-157X4.

### Generation of HIV-1 mutant strains.

To insert the Pol266I mutation into the VI-157X4 plasmid, the plasmid was amplified using by PrimeSTAR HS DNA polymerase (TaKaRa) with overlapping primers that recognized the target mutation. The fragment of the *pol* region between ApaI and AflII sites was subsequently amplified by using TaKaRa *Ex Taq* (TaKaRa) and then ligated into a pCR2.1 vector (Invitrogen) by TA cloning. After TA cloning of the *pol* fragment, the plasmids were digested with ApaI and AflII, and the 2.7-kb fragment was purified and ligated into the ApaI-AflII-digested site in VI-157X4 plasmids. Finally, insertion of the Pol266I mutation into the VI-157X4 plasmid was confirmed by sequencing the region including Pol266. To obtain mutant viruses, we transfected 293T cells with VI-157X4 plasmids including each mutation using Lipofectamine 2000 (Invitrogen).

### Expansion of HIV-1-specific bulk T cells and ICS assay.

PBMCs from HLA-B*15:02^+^ individuals were stimulated with 1 μM 17-mer peptide pools or epitope peptides and then cultured for 12 to 14 days in RPMI 1640 medium (Thermo Fisher Scientific) containing 10% fetal calf serum (FCS), 20 ng/mL human recombinant interleukin 2 (rIL-2) (ProSpec, Ness Ziona, Israel), 1× minimum essential medium (MEM) nonessential amino acids solution (Gibco, Grand Island, NY, USA), and 1 mM sodium pyruvate solution (Gibco). .221-B*15:02 cells, .221-B*15:02 cells prepulsed with peptides, or 721.221 cells infected with VI-157X4 were cocultured with bulk T cells in a 96-well plate for 2 h at 37°C. Brefeldin A (10 μg/mL) was then added, and the cells were incubated for 4 h at 37°C. The cells were then fixed with 4% paraformaldehyde and permeabilized with saponin buffer (0.1% saponin–10% FBS–phosphate-buffered saline [PBS]) after staining with allophycocyanin (APC)-labeled anti-CD8 monoclonal antibodies (MAb) (BioLegend, San Diego, CA, USA). Next, the cells were stained with a phycoerythrin (PE)-labeled anti-gamma interferon (anti-IFN-γ) MAb (BioLegend). The percentage of IFN-γ-producing cells among the CD8^+^ T cell population was analyzed by FACS Canto II (BD Biosciences, Franklin Lakes, NJ, USA) and FlowJo 10.7.1 software.

### IFN-γ ELISpot assay.

ELISpot assays were performed as previously described (18). Briefly, 1 × 10^5^ PBMCs from each individual were plated into each well of a 96-well polyvinylidene difluoride plate (Millipore) that had been precoated with 5 μg/mL anti-IFN-γ MAb 1-D1K (Mabtech, Stockholm, Sweden) at a concentration of 1 μM for each peptide. The plates were then incubated for 16 h at 37°C and subsequently washed with PBS before the addition of biotinylated anti-IFN-γ MAb (Mabtech) at 1 μg/mL. After the plates had been incubated for 90 min at room temperature, they were washed with PBS and then incubated with streptavidin-conjugated alkaline phosphatase (Mabtech) for 60 min at room temperature. Following washes with PBS, individual cytokine-producing cells were visualized as dark spots after a 20-min reaction with 5-bromo-4-chloro-3-indolylphosphate and nitroblue tetrazolium in the presence of an alkaline phosphatase-conjugated substrate (Bio-Rad, Hercules, CA, USA). The spots were counted with an Eliphoto-Counter (Minerva Teck, Kanagawa, Japan). The number of spots was calculated per 10^6^ CD8^+^ T cells or 10^6^ PBMCs. The number of spots for each peptide-specific T cell response was finally calculated by subtracting the number of spots in wells without peptides: 150 spots per 10^6^ CD8^+^ T cells and 100 spots per 10^6^ PBMCs were defined as positive responses to HIV-1 peptide cocktails and HIV-1 epitope peptides, respectively, based on the analysis of responses to HIV-1 peptides using PBMCs from HIV-1-naive individuals. The mean values + 5 standard deviations (SD) of the spot-forming units (SFU) of samples from 12 HIV-1-naive individuals for the peptide pool were 115 SFU/10^6^ CD8^+^ T cells and 46 SFU/10^6^ PBMCs in a previous study (27).

### Statistical analysis.

The Mann-Whitney U test and unpaired *t* test were performed to compare two groups in this study. Correlations between the breadth of T cell responses and pVL were statistically analyzed using Spearman’s rank test. The frequency of mutations between HLA-B*15:02^+^ and HLA-B*15:02^−^ individuals was analyzed statistically using Fisher’s exact test. A *P* value of <0.05 was considered statistically significant.
